# Semaglutide and Follow-On Peptide Therapeutics: Balancing Innovation, Regulation, and Clinical Outcomes

**DOI:** 10.7759/cureus.108999

**Published:** 2026-05-16

**Authors:** Brij Teli, Shrikant Somani, Vivek Arya, Prashant J Rajput, Pravin Kahale, Vahid Bharmal, Shalin Shah, Vivek Patel, Anand Ahuja, Sonali Patange

**Affiliations:** 1 Endocrinology, Diabetes, and Metabolism, Granthi- Diabetes, Endocrine and Obesity Center, A Unit of “Aumkar Clinic”, Rajkot, IND; 2 Endocrinology and Diabetes, Somani Diabetes and Endocrine Care Center, Ahmedabad, IND; 3 Endocrinology and Diabetes, Center for Endocrine Disease and Diabetes, Ahmedabad, IND; 4 Nephrology, Gleneagles Hospital, Mumbai, IND; 5 Cardiology, Kokilaben Hospital, Mumbai, IND; 6 Endocrinology, Bhailal Amin General Hospital, Vadodara, IND; 7 Endocrinology and Diabetes, Gujarat Diabetes and Endocrine Foundation, Ahmedabad, IND; 8 Endocrinology, Marengo CIMS Hospital, Ahmedabad, IND; 9 Cardiovascular Surgery, Rhythm Heart Institute, Vadodara, IND; 10 Diabetes and Endocrinology, Speciality Diabetes Clinic, Mumbai, IND

**Keywords:** clinical outcomes, obesity, originator, peptide, recombinant, semaglutide, synthetic, type 2 diabetes mellitus

## Abstract

Glucagon-like peptide-1 receptor agonists (GLP-1 RAs) have emerged as an important therapeutic class for the management of type 2 diabetes mellitus (T2DM) and obesity owing to their ability to improve glycemic control, promote weight loss, reduce cardiometabolic parameters, and have the added advantage of a low risk of hypoglycemia due to glucose-independent insulin secretion. Semaglutide, a long-acting GLP-1 RA developed using a semi-recombinant process, has demonstrated significant benefits across metabolic and cardiovascular outcomes. Amid the increasing need for affordable therapies, there has been a rising interest in developing synthetic peptide versions of glucagon-like peptide-1 (GLP-1)-based drugs originally produced using recombinant deoxyribonucleic acid (rDNA) technology. However, the transition from recombinant production to chemical peptide synthesis raises important considerations about regulatory pathways, manufacturing processes, and potential clinical implications. Regulatory approaches for synthetic peptides vary across regions. The European Union and the United States follow distinct frameworks, with India adopting a risk-based regulatory approach that may require extensive quality, safety, and clinical data. Manufacturing processes can influence impurity profiles, stability, and immunogenicity, potentially affecting clinical outcomes. Therefore, the approval of follow-on versions of semaglutide requires a rigorous demonstration of both quality and clinical comparability with the originator, consistent with principles applied to biosimilars. Online databases such as PubMed, Scopus, and Google Scholar were used to source relevant research articles. This narrative review aims to discuss the development of semaglutide, examine global and national regulatory perspectives on synthetic and recombinant peptides, and explore the clinical implications of manufacturing variability. It also highlights the expanding therapeutic potential of semaglutide beyond obesity and T2DM.

## Introduction and background

Introduction

During the lifecycle of a biopharmaceutical product, manufacturers may develop follow-on versions that refer to an already approved product, known as the reference product. Semaglutide, a polypeptide analog of human glucagon-like peptide-1 (GLP-1), lowers blood glucose by stimulating insulin secretion and suppressing glucagon release [1]. Semaglutide (Ozempic®) was approved by the US Food and Drug Administration (FDA) in December 2017 and the European Medicines Agency (EMA) in February 2018 for the treatment of type 2 diabetes mellitus (T2DM). Following this, semaglutide was also approved for weight management in adults with obesity or overweight with comorbid conditions (Wegovy®) by the FDA in June 2021 and the EMA in January 2022 [2].

With expanding indications and increasing global demand, follow-on and compounded semaglutide products have emerged in several markets. However, variations in manufacturing methods and process controls may introduce new impurities or increase the levels of known impurities in the drug substance and final product [1].

Peptide therapeutics pose regulatory challenges due to their intermediate molecular complexity, susceptibility to degradation, potential immunogenicity, and sensitivity to manufacturing changes. They may exhibit structural heterogeneity such as sequence truncations, racemization, aggregation, and post-synthetic modifications. At the same time, peptides often lack the higher-order structural complexity typical of biologics, making regulatory classification challenging. Consequently, regulatory approaches vary across jurisdictions, particularly regarding product classification; Chemistry, Manufacturing, and Controls (CMC) requirements; and clinical development pathways [3].

To ensure the safety and efficacy of follow-on polypeptides, regulatory agencies, including the FDA, the EMA, and the Central Drugs Standard Control Organization (CDSCO) in India, have established frameworks to evaluate similarity with reference products [1,3]. This narrative review examines and compares the regulatory frameworks governing peptide therapeutics in the United States, the European Union, and India and highlights the therapeutic potential of semaglutide beyond obesity and T2DM.

Materials and methods

Electronic databases, including PubMed, Scopus, and Google Scholar, were systematically searched to identify relevant literature on GLP-1 receptor agonists (GLP-1 RAs), semaglutide, peptide therapeutics, manufacturing technologies, regulatory pathways, and clinical outcomes. The search strategy included combinations of the following keywords: GLP-1 RAs, semaglutide, peptide therapeutics, recombinant deoxyribonucleic acid (rDNA) technology, synthetic peptides, follow-on peptides, reference listed drugs (RLDs), Abbreviated New Drug Application (ANDA), New Drug Application (NDA), biosimilars, immunogenicity, manufacturing processes, impurity profiles, regulatory framework, cardiovascular outcomes, obesity, and type 2 diabetes mellitus (T2DM). Only articles published in the English language and publications from the last 15 years addressing the development, manufacturing technologies, regulatory pathways, and clinical outcomes of GLP-1 RAs were considered.

## Review

GLP-1 RAs and the original development of semaglutide

GLP-1 RAs are widely used in the management of T2DM because they effectively improve glycemic control, promote weight reduction, lower blood pressure, and have a low risk of causing hypoglycemia [4]. Semaglutide is a long-acting GLP-1 RA indicated for the treatment of T2DM, obesity, and obesity-related complications. Advances in peptide synthesis and analytical technologies have facilitated the development of synthetic generic peptides corresponding to RLDs that were originally produced using rDNA technology [5]. Developed by Novo Nordisk, semaglutide received approval from the FDA in 2017 [6]. The commercial production of semaglutide utilizes a hybrid manufacturing approach that integrates recombinant expression technology with subsequent synthetic modification steps to incorporate the N-terminal oligopeptide and the C18 fatty acid side chain. This strategy combines the efficiency of chemical peptide synthesis with the economic advantages of biotechnological production [7]. Because the originator product is manufactured using rDNA technology, considerable interest has emerged in developing fully synthetic peptide versions as potential generic alternatives [5].

Regulatory approaches to follow-on peptides in the United States, the EU, and India

The growing demand for affordable and effective therapies, particularly for widely used peptide drugs such as Ozempic® and Wegovy®, has led to increased efforts to develop generic alternatives to address perceived shortages of RLDs [8]. To ensure that follow-on polypeptides maintain comparable safety and efficacy to their reference products, regulatory frameworks have been established by agencies such as the EMA and the FDA to assess the similarity of these products [1]. To facilitate access to lower-cost generics, the FDA introduced the ANDA pathway, which streamlines the approval process while maintaining strict standards for quality, safety, and efficacy [8]. However, the FDA has indicated that an ANDA may not provide sufficient evidence, such as clinical data evaluating potential immunogenicity, for the approval of peptides derived from rDNA technology. In such cases, applicants may need to pursue approval through a 505(b)(2) application or a stand-alone NDA under section 505(b)(1) of the Federal Food, Drug, and Cosmetic Act [9]. The FDA typically classifies chemically synthesized peptides, particularly those containing fewer than 40 amino acids, as small-molecule drugs, allowing them to be evaluated through NDA pathways such as 505(b)(1) or 505(b)(2) [3].

The emergence of synthetic peptide manufacturing has also influenced regulatory considerations in the European Union (EU). While biosimilars are evaluated under a dedicated regulatory framework, synthetically produced follow-on polypeptides referencing biologic originators are generally not eligible for the biosimilar pathway and instead must have approval through the traditional generic pathway under Article 10(1) or the hybrid pathway under Article 10 (3). Recombinant DNA technology has significantly shaped the pharmaceutical landscape over the past four decades, with hundreds of recombinant biologics approved in the EU since 1988. To promote competition in this sector, the EU established a dedicated biosimilar regulatory pathway in 2005 under Article 10(4) of Directive 2001/83/EC, enabling the rigorous evaluation of biologic follow-on products while accounting for their complexity [10].

In India, peptide therapeutics are regulated by the Central Drugs Standard Control Organization (CDSCO) [3]. The office of the Drugs Controller General of India (DCGI) is responsible for ensuring that generic products demonstrate safety, tolerability, and comparability with their innovator counterparts before approval for human use. Regulatory requirements include bioavailability and bioequivalence studies to confirm therapeutic equivalence between test products and reference drugs [11]. Unlike the FDA’s size-based classification of peptides, CDSCO generally follows a more process-oriented and risk-based regulatory approach, often applying scrutiny similar to that used for biologics, regardless of peptide length. This may involve extensive preclinical safety data; detailed Chemistry, Manufacturing, and Control (CMC) documentation; and, in some cases, local clinical trial requirements. The lack of peptide-specific regulatory guidance in India can also lead to variability in interpretation, increasing development challenges for both domestic manufacturers and multinational companies [3]. A comparative overview of regulatory frameworks across the United States, the EU, and India highlights key differences in authority structure, classification criteria, and approval pathways for peptide-based therapeutics (Table 1) [3,10,12-15].

**Table 1:** Comparative analysis of regulatory frameworks in the United States, the EU, and India Source: [3,10,12-15] FDA, Food and Drug Administration; EMA, European Medicines Agency; EU, European Union; CDSCO, Central Drugs Standard Control Organization; CHMP, Committee for Medicinal Products for Human Use; CDER, Center for Drug Evaluation and Research; ANDA, Abbreviated New Drug Application; NDA, New Drug Application; FD&C, Federal Food, Drug, and Cosmetic Act; DCGI, Drugs Controller General of India; NDCT, New Drugs and Clinical Trials; EC, European Commission; PHS, Public Health Service

Regulatory Aspect	FDA	EMA	CDSCO
Regulatory authority	US FDA, mainly through CDER	EMA via CHMP and national competent authorities	CDSCO under DCGI
Governing legislation	FD&C Act; PHS Act (limited cases)	Regulation (EU) No. 2019/6, Regulation (EC) No. 726/2004, and Directive 2001/83/EC	Drugs and Cosmetics Act, 1940; NDCT Rules, 2019
Product classification criteria	Molecule-centric and risk-based; chemically synthesized peptides (<40 amino acids) usually regarded as small molecules	Molecule-centric	Process-centric and conservative; peptides often regulated under biologics-like frameworks, irrespective of size
Marketing authorization pathways	NDA (505(b)(1)), NDA (505(b)(2)), and ANDA for follow-on peptides	Directive 2001/83/EC	New Drug Application under NDCT Rules, 2019
Follow-on/generic peptides	Streamlined ANDA pathway based on pharmaceutical equivalence and bioequivalence	Article 10(4) of Directive 2001/83/EC for biosimilar; Article 10(1) of Directive 2001/83/EC for generic	No clearly established abbreviated pathway; frequently treated in a manner similar to biosimilars

Impact of manufacturing processes on peptide drug quality and immunogenicity

**Figure 1:**
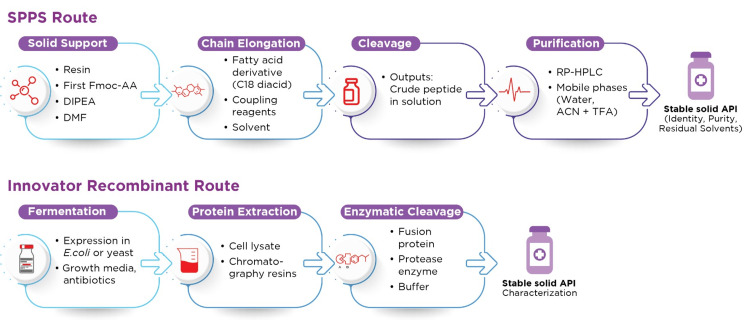
The manufacturing paradigm: synthetic versus recombinant route Source: [16,17]. The image was created by Adobe Illustrator (Adobe Inc., Mountain View, CA) SPPS, solid-phase peptide synthesis; Fmoc, 9-fluorenylmethyloxycarbonyl; AA, amino acid; DIPEA, N,N-diisopropylethylamine; DMF, dimethylformamide; RP-HPLC, reverse-phase high-performance liquid chromatography; ACN, acetonitrile; TFA, trifluoroacetic acid; *E. coli*, *Escherichia coli*; API, active pharmaceutical ingredient

Most originator peptide therapeutics have traditionally been produced using rDNA technology, whereas most follow-on semaglutide products are developed using total chemical synthesis, wherein the peptide is assembled amino acid by amino acid through solid-phase peptide synthesis (SPPS), followed by the chemical attachment of the fatty acid side chain. These alternative manufacturing approaches may alter the clinical characteristics of the product (Figure 1) [16,17]. Even minor modifications in the manufacturing process can significantly influence the chemical and physical stability of the drug, as well as its impurity profile, which may in turn affect its immunogenic potential [18].

Although the amino acid sequence of the active pharmaceutical ingredient (API) may remain identical to that of the originator product, differences in production methods, such as transitioning from recombinant expression to chemical synthesis, can introduce structural variations, including additional or duplicated amino acids or side-chain modifications. These alterations may generate impurities that are difficult to separate from the API and may potentially affect the clinical performance of the drug, including its immunogenicity [16]. Semaglutide, developed by Novo Nordisk, is manufactured using a hybrid process that combines microbial fermentation with chemical synthesis [7]. These multistep processes were specifically designed for semaglutide and are closely monitored and controlled to ensure consistent product quality. Given the complexity of this approach and the potential risk for impurities, stringent quality control measures are in place to ensure product safety, efficacy, and consistency. Differences in manufacturing processes, scale and production equipment, and the choice of host cell and raw materials mean that follow-on versions of liraglutide and semaglutide may have physical and chemical differences compared with the originator products (Table 2) [1,4,5,7,16,19,20]. Furthermore, a recent case study evaluating impurities in liraglutide and semaglutide products demonstrated that the same peptide produced by different manufacturers and through different manufacturing techniques (recombinant versus synthetic) can exhibit markedly distinct impurity profiles [16]. Such variations were also observed by Hach et al. in tested samples of follow-on injectable semaglutide and liraglutide, as well as oral semaglutide (versus originators), which showed differences in impurity composition, potential stability concerns, reduced API content, and possible immunogenic risks. The clinical implications of these differences for patients receiving follow-on products may require further evaluation through dedicated clinical studies. In addition, findings from certain samples suggested deficiencies in Good Manufacturing Practice (GMP) compliance at the manufacturing sites. These observations highlight the need for the rigorous regulatory assessment of follow-on GLP-1 analogs, including compounded formulations, to ensure their quality, safety, and efficacy [1].

**Table 2:** Difference between innovator (rDNA) and synthetic semaglutide Source: [1,4,5,7,16,19,20] rDNA, recombinant deoxyribonucleic acid; NDA, New Drug Application; PK, pharmacokinetics

Parameter	Innovator (rDNA) Semaglutide	Synthetic Semaglutide
Heritage	Since 2017	2024/25
How is it made?	Hybrid approach, combining recombinant expression technology with synthetic modification steps to attach an N-terminal oligopeptide and a C18 fatty acid motif	Stepwise coupling of amino acids, linker, and fatty chain
Sequence fidelity	Peptide backbone produced by rDNA, therefore accurate	Coupling errors, truncations, and deletions possible
Critical modifications	Precisely inserted and validated	Higher risk of misacylation or missing groups
Impurity profile	Low, consistent, and NDA-qualified	Multiple peptide-related and unknown impurities reported
Stability and aggregation	Optimized, long-acting PK	Higher risk of aggregation and degradation
Immunogenicity risk	Minimal risk	Elevated risk due to impurities and aggregates
Patient experience	>49 million patients-years of exposure	Very limited exposure

**Figure 2:**
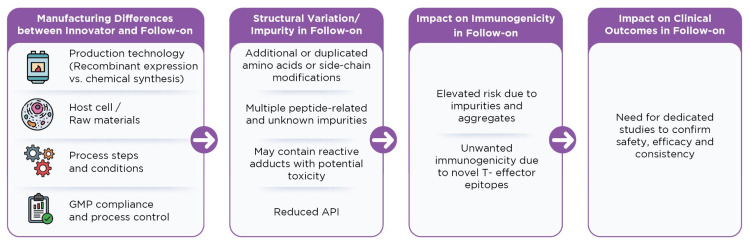
Impact of manufacturing differences on structure, impurity, immunogenicity, and clinical outcomes Source: [1,7,16,18]. The image was created by Adobe Illustrator API, active pharmaceutical ingredient; GMP, Good Manufacturing Practice

Recombinant expression provides tighter control over molecular integrity, impurity profile, and pharmacokinetic-critical modifications, which are important for a lipidated, long-acting peptide such as semaglutide [1,5]. This section underscores that the clinical efficacy and safety of semaglutide are intrinsically linked to the quality of its manufacturing. Even subtle variations in molecular structure, impurity profile, or stability can impact pharmacokinetics, immunogenicity, and overall therapeutic outcomes (Figure 2) [1,7,16,18]. Therefore, stringent and consistent manufacturing processes are crucial to ensuring the reliable clinical performance demonstrated in subsequent clinical studies.

Addressing obesity in Asian populations

According to recommendations from the National Institute for Health and Care Excellence (NICE) and various global guidelines, individuals of South Asian, Chinese, other Asian, Middle Eastern, Black African, or African-Caribbean origin are more likely to develop central adiposity and experience cardiometabolic risk at lower body mass index (BMI) levels. Therefore, lower BMI thresholds are suggested to define overweight and obesity in these populations, with overweight classified as a BMI of 23-27.4 kg/m² and obesity as a BMI of 27.5 kg/m² or higher [21]. South Asian adults, in particular, have a greater burden of cardiovascular risk factors at a given BMI compared to White Caucasian populations. This increased risk is largely attributed to a higher percentage of body fat for the same BMI and a greater tendency to accumulate abdominal fat [22].

In Asian individuals with obesity (BMI ≥ 25 kg/m²), treatment with the once-weekly injectable semaglutide 2.4 mg has been shown to produce significant reductions in body weight while maintaining good tolerability. These findings have important clinical and public health implications for Asian countries, where obesity is defined at lower BMI thresholds than in Western populations. The demonstrated efficacy and safety of injectable semaglutide 2.4 mg support its consideration for inclusion in regional treatment guidelines [23]. Additionally, studies in East Asian adults with obesity, with or without T2DM, demonstrate that once-weekly semaglutide 2.4 mg produces substantial and clinically meaningful reductions in body weight and abdominal visceral fat compared to placebo. By week 68, the estimated mean reduction in body weight was 13.2% with semaglutide versus 2.1% with placebo, corresponding to a significant estimated treatment difference (ETD) of -11.1 percentage points (p < 0.0001). Importantly, semaglutide 2.4 mg also led to a pronounced reduction in the abdominal visceral fat area (40.0%) compared to 6.9% in the placebo group [24]. The efficacy of semaglutide in Asian populations was further supported by the SURMOUNT-5 trial, where, at week 72, semaglutide 2.4 mg achieved a 20.6% reduction in body weight compared to 19.2% with tirzepatide [25].

Another recent real-world study by Shah et al. demonstrated that the injection of semaglutide 2.4 mg for six months resulted in significant reductions in weight, BMI, and body fat percentage. Mean body weight decreased by 13.39 ± 6.04 kg (ETD), corresponding to a 14.7% relative reduction from baseline (p < 0.001), highlighting its robust effectiveness in real-world clinical practice [26].

Innovator versus generic semaglutide: Beyond obesity outcomes

Beyond weight management and T2DM, the innovator semaglutide (Wegovy®) clinical program, including SELECT and STEP trials, has demonstrated a broad spectrum of benefits. These include significant cardiovascular risk reduction, improved outcomes in heart failure with preserved ejection fraction, renal protection, and favorable effects on metabolic parameters. Additionally, benefits have been observed in metabolic dysfunction-associated steatohepatitis and obesity-related osteoarthritis, underscoring its multidimensional therapeutic potential beyond glycemic control and weight loss [2].

However, it is important to note that phase 3 non-inferiority trials comparing innovator with generic semaglutide have primarily been designed and evaluated based on obesity and diabetes management endpoints, without adequately assessing these broader cardiometabolic and organ-protective benefits [27,28].

Multiorgan and musculoskeletal protection with semaglutide

Cardiovascular Protection

Overweight and obesity are independent risk factors for cardiovascular events. In the SELECT trial, in a cohort of 17,604 patients with a BMI ≥ 27 kg/m² and established cardiovascular disease but without diabetes, once-weekly subcutaneous semaglutide 2.4 mg administered over a mean duration of 33 months resulted in a 20% reduction in the risk of major adverse cardiovascular events (MACE), including cardiovascular death, nonfatal myocardial infarction, and nonfatal stroke. In subgroup analyses, the reduction in cardiovascular risk with semaglutide was most pronounced in the Asian population (hazard ratio {HR}: 0.64), followed by White individuals (HR: 0.81) and Black/African individuals (HR: 0.87) [29].

A post hoc analysis combining data from the Semaglutide Unabated Sustainability in Treatment of Type 2 Diabetes (SUSTAIN) 6 and Peptide Innovation for Early Diabetes Treatment (PIONEER) 6 cardiovascular outcome trials (CVOTs) further showed that semaglutide reduced the risk of MACE by 24% compared to placebo in patients with T2DM. This benefit was consistent across both subcutaneous and oral formulations of semaglutide and across several clinically relevant subgroups, including individuals with or without established cardiovascular disease or chronic kidney disease, as well as those with or without a prior history of myocardial infarction or stroke [30].

Nephroprotection

Patients with T2DM and chronic kidney disease are at an increased risk for kidney failure and cardiovascular complications. Therefore, evaluating the potential benefits and safety of glucose-lowering therapies in this population is essential to reduce mortality and prevent kidney and cardiovascular outcomes [31]. In a study by Perkovic et al., treatment with injectable semaglutide 1.0 mg reduced the risk of the primary kidney composite outcome event by 24% compared with placebo (331 versus 410 first events; p = 0.0003). Semaglutide showed favorable secondary outcomes, including a slower decline in renal function, reflected by a less steep annual estimated glomerular filtration rate (eGFR) slope by 1.16 mL/minute/1.73 m² (p < 0.001), an 18% lower risk of major cardiovascular events (p = 0.029), and a 20% reduction in all-cause mortality (p = 0.01) [32].

Hepatoprotection

In a phase 3, multicenter, randomized, double-blind, placebo-controlled trial, the resolution of steatohepatitis without fibrosis worsening was achieved in 62.9% of patients receiving injectable semaglutide 2.4 mg compared to 34.3% with placebo (p < 0.001). Additionally, improvement in liver fibrosis without worsening steatohepatitis was observed in 36.8% of the semaglutide group compared to 22.4% in the placebo group (p < 0.001) [33].

Supporting these findings, a real-world study by Suki et al. demonstrated that semaglutide significantly improved liver enzymes and hepatic synthetic function in patients with metabolic dysfunction-associated steatotic liver disease (MASLD), indicating reduced inflammation and preserved liver function. The study also reported a lower risk of progression to advanced liver disease among semaglutide users, highlighting its potential hepatoprotective benefits [34].

Cognitive Protection

Both T2DM and obesity are important modifiable risk factors for Alzheimer’s disease (AD). Semaglutide has been shown to improve cardiovascular health, alcohol use, smoking, and depression, factors linked to AD risk. In patients with T2DM, semaglutide was associated with a 40%-70% lower risk of first-time AD diagnosis compared to other antidiabetic drugs, including other GLP-1 RAs, along with reduced AD-related medication use. These real-world findings support its potential role in reducing the onset and progression of AD [35].

Musculoskeletal Benefit

Obesity is also a key contributor to the development and progression of knee osteoarthritis. Among individuals with obesity and moderate-to-severe knee osteoarthritis pain, once-weekly injectable semaglutide 2.4 mg produced significantly greater reductions in osteoarthritis-related pain score by 41.7 points versus 27.5 points with placebo [36].

## Conclusions

Semaglutide represents a paradigm shift in the management of metabolic diseases, extending well beyond glycemic control to deliver broad, clinically significant, weight-loss-independent benefits across multiple organ systems. Robust evidence from clinical trials and real-world studies demonstrates its efficacy in reducing cardiovascular risk, preserving renal function, improving hepatic outcomes, and potentially lowering the risk of neurodegenerative conditions such as AD. Importantly, its efficacy and tolerability in Asian populations, where cardiometabolic risk occurs at lower BMI, support its relevance in regional practice.

Semaglutide, the originator, is manufactured using a hybrid process that combines microbial fermentation with solid-phase peptide synthesis (SPPS). This manufacturing strategy integrates recombinant peptide expression with subsequent chemical modifications to generate the final molecule. Given the complexity of this approach and the potential risk for impurities, stringent quality control measures are implemented throughout the production process to ensure product safety, efficacy, and batch-to-batch consistency. Interest in developing cost-effective synthetic generic versions of semaglutide has increased in recent years. However, peptide therapeutics pose unique regulatory and manufacturing challenges. Variations in production methods, including recombinant and chemical synthesis techniques, may alter impurity profiles, stability, and immunogenic potential, thereby necessitating rigorous quality assessment. Consequently, robust regulatory frameworks are essential to ensure the safety, quality, and therapeutic equivalence of follow-on peptide products. Careful evaluation through stringent analytical, manufacturing, and clinical standards is therefore crucial to safeguard patient outcomes while enabling broader access to these important therapeutics.

## References

[1] Hach M, Engelund DK, Mysling S (2024). Impact of manufacturing process and compounding on properties and quality of follow-on GLP-1 polypeptide drugs. Pharm Res.

[2] Salvador R, Moutinho CG, Sousa C, Vinha AF, Carvalho M, Matos C (2025). Semaglutide as a GLP-1 agonist: a breakthrough in obesity treatment. Pharmaceuticals (Basel).

[3] Praveen RB, Raghavan V, Iswareyaa RA (2026). A comprehensive review of regulatory requirements and approval pathways for peptide therapeutics in the USA and India. Int J Pharm Sci.

[4] Liu X, Zhang N, Gu X, Qin Y, Song D, Zhang L, Ma S (2020). Total synthesis of semaglutide based on a soluble hydrophobic-support-assisted liquid-phase synthetic method. ACS Comb Sci.

[5] Kim SH, Kim SS, Kim HJ, Park EJ, Na DH (2025). Peptide mapping analysis of synthetic semaglutide and liraglutide for generic development of drugs originating from recombinant DNA technology. J Pharm Biomed Anal.

[6] Al Musaimi O (2024). Exploring FDA-approved frontiers: insights into natural and engineered peptide analogues in the GLP-1, GIP, GHRH, CCK, ACTH, and α-MSH realms. Biomolecules.

[7] Parisis V, da Gama Ferreira R, Misailidis N (2025). Production of semaglutide: process modeling and cost analysis using SuperPro Designer®. https://www.researchgate.net/profile/Demetri-Petrides/publication/392573822_Semaglutide_Ozempic_and_Wegovy_Manufacturing_-_Process_Modeling_and_Techno-Economic_Assessment_TEA_using_SuperPro_Designer/links/68493bb0c33afe388acb4d95/Semaglutide-Ozempic-and-Wegovy-Manufacturing-Process-Modeling-and-Techno-Economic-Assessment-TEA-using-SuperPro-Designer.pdf.

[8] De Groot AS, Mattei A, Gabriel B (2025). Immunogenicity of generic peptide impurities: current orthogonal approaches. Pharm Res.

[9] (2026). ANDAs for certain highly purified synthetic peptide drug products that refer to listed drugs of rDNA origin guidance for industry. https://www.fda.gov/regulatory-information/search-fda-guidance-documents/andas-certain-highly-purified-synthetic-peptide-drug-products-refer-listed-drugs-rdna-origin.

[10] Klein K, Heisterberg J, Stolk P (2024). Synthetic polypeptides using a biologic as a reference medicinal product - the European landscape of regulatory approvals. Front Med (Lausanne).

[11] (2026). Guidance document for BA/BE NOC for export. https://cdsco.gov.in/opencms/export/sites/CDSCO_WEB/Pdf-documents/BA_BE/guidance_doc_BABE_NOC1_1Jan2018.pdf.

[12] (2026). How the committees work. https://www.ema.europa.eu/en/committees/how-committees-work.

[13] (2026). Legal framework. https://www.ema.europa.eu/en/about-us/what-we-do/legal-framework.

[14] (2026). Guideline on similar biological medicinal products. https://www.ema.europa.eu/en/documents/scientific-guideline/guideline-similar-biological-medicinal-products-rev1_en.pdf.

[15] (2026). QRD general principles regarding the SmPC information for a generic/hybrid/biosimilar product. https://www.ema.europa.eu/en/documents/regulatory-procedural-guideline/quality-review-documents-general-principles-regarding-summary-product-characteristics-information-generic-hybrid-biosimilar-product_en.pdf.

[16] De Groot AS, Roberts BJ, Mattei A, Lelias S, Boyle C, Martin WD (2023). Immunogenicity risk assessment of synthetic peptide drugs and their impurities. Drug Discov Today.

[17] (2026). Generic peptide drugs: biosimilar pipeline. https://www.peptidejournal.org/news/generic-peptide-drugs-biosimilar-pipeline.

[18] Staby A, Steensgaard DB, Haselmann KF (2020). Influence of production process and scale on quality of polypeptide drugs: a case study on GLP-1 analogs. Pharm Res.

[19] (2026). Citizen petition requesting regulatory action concerning compounded semaglutide preparations. https://share.google/obNePbbCaZiWNtD2q.

[20] (2026). Novo Nordisk - a focused healthcare company: investor presentation first three months of 2025. https://www.novonordisk.com/content/dam/nncorp/global/en/investors/pdfs/financial-results/2025/Q1-2025-investor-presentation.pdf.

[21] (2026). Overweight and obesity management. https://www.nice.org.uk/guidance/ng246/chapter/Identifying-and-assessing-overweight-obesity-and-central-adiposity.

[22] Wahi G, Anand SS (2013). Race/ethnicity, obesity, and related cardio-metabolic risk factors: a life-course perspective. Curr Cardiovasc Risk Rep.

[23] Lim S, Buranapin S, Bao X (2025). Once-weekly semaglutide 2·4 mg in an Asian population with obesity, defined as BMI ≥25 kg/m2, in South Korea and Thailand (STEP 11): a randomised, double-blind, placebo-controlled, phase 3 trial. Lancet Diabetes Endocrinol.

[24] Kadowaki T, Isendahl J, Khalid U (2022). Semaglutide once a week in adults with overweight or obesity, with or without type 2 diabetes in an east Asian population (STEP 6): a randomised, double-blind, double-dummy, placebo-controlled, phase 3a trial. Lancet Diabetes Endocrinol.

[25] Aronne LJ, Horn DB, le Roux CW (2025). Tirzepatide as compared with semaglutide for the treatment of obesity. N Engl J Med.

[26] Shah R, Unadkat V, Parekh M (2026). Real-world evidence of injection semaglutide 2.4 mg in Indian adults with obesity: effects on weight loss and metabolic outcomes. Int J Sci Stud.

[27] Sharma PK, Redkar SV, Madhav Karmalkar A (2026). Efficacy and safety of semaglutide injection in comparison with reference semaglutide for chronic weight management in indian adults with obesity: a phase III randomized non-inferiority trial. Metabol Open.

[28] Kapoor N, Kalra S, Naskar A (2026). Comparative efficacy and safety of once-weekly semaglutide formulations in Indian adults with obesity: a phase III, randomized non-inferiority active-controlled study (size plus study). Cureus.

[29] Lincoff AM, Brown-Frandsen K, Colhoun HM (2023). Semaglutide and cardiovascular outcomes in obesity without diabetes. N Engl J Med.

[30] Husain M, Bain SC, Jeppesen OK, Lingvay I, Sørrig R, Treppendahl MB, Vilsbøll T (2020). Semaglutide (SUSTAIN and PIONEER) reduces cardiovascular events in type 2 diabetes across varying cardiovascular risk. Diabetes Obes Metab.

[31] Mann JF, Rossing P, Bakris G (2024). Effects of semaglutide with and without concomitant SGLT2 inhibitor use in participants with type 2 diabetes and chronic kidney disease in the FLOW trial. Nat Med.

[32] Perkovic V, Tuttle KR, Rossing P (2024). Effects of semaglutide on chronic kidney disease in patients with type 2 diabetes. N Engl J Med.

[33] Sanyal AJ, Newsome PN, Kliers I (2025). Phase 3 trial of semaglutide in metabolic dysfunction-associated steatohepatitis. N Engl J Med.

[34] Suki M, Amer J, Milgrom Y (2025). Semaglutide in MASLD patients: improved survival and liver outcomes. Pharmaceuticals (Basel).

[35] Wang W, Wang Q, Qi X (2024). Associations of semaglutide with first-time diagnosis of Alzheimer's disease in patients with type 2 diabetes: target trial emulation using nationwide real-world data in the US. Alzheimers Dement.

[36] Bliddal H, Bays H, Czernichow S (2024). Once-weekly semaglutide in persons with obesity and knee osteoarthritis. N Engl J Med.

